# Fisetin Attenuates Lipopolysaccharide-Induced Inflammatory Responses in Macrophage

**DOI:** 10.1155/2021/5570885

**Published:** 2021-04-13

**Authors:** Yoshiko Hada, Haruhito A. Uchida, Jun Wada

**Affiliations:** ^1^Department of Nephrology, Rheumatology, Endocrinology and Metabolism, Okayama University Graduate School of Medicine, Dentistry and Pharmaceutical Science, Okayama, Japan; ^2^Department of Chronic Kidney Disease and Cardiovascular Disease, Okayama University Graduate School of Medicine, Dentistry and Pharmaceutical Science, Okayama, Japan

## Abstract

Several studies have reported the efficacy and safety of polyphenols in human health; however, the verification of their efficacy remains insufficient. The aim of this study was to examine whether fisetin, one of flavonoids prevalently present in fruits and vegetables, could suppress lipopolysaccharide- (LPS-) induced inflammatory responses in macrophages. LPS increased proinflammatory mRNA abundance (MCP 1, IL-1*β*, and iNOS) but were suppressed by fisetin. The increment of nitric oxide by LPS, an oxidative stress factor, was attenuated by fisetin. In addition, LPS-enhanced phosphorylation of mitogen-activated protein kinase (ERK and JNK) was reduced. Finally, fisetin attenuated the expression or activity of uPA, uPAR, MMP-2, and MMP-9, which are known as associated factors of macrophage recruitment or infiltration. In conclusion, fisetin is a promising therapeutic agent for macrophage-related inflammation diseases, like sepsis and atherosclerosis.

## 1. Introduction

The therapeutic usage of natural components has gained more attention in the last 2 decades. Based on their great efficacy and low toxicity, 40% of FDA-approved therapeutic agents are natural-based components or their derivatives [1]. Toxicity associated with other drugs has been successfully prevented by different natural products [2]. Thus, it is very important and significant to explore plant-derived natural compounds further.

Polyphenols are the plant secondary metabolites and widely present in foods and beverages of plant origin, fruits, vegetables, spices, tea, and wine. Although they are not considered as essential micronutrients, several literatures evince their beneficial effects on human health, especially in diets associated with high consumption of fruits and vegetables [3–5]. Numerous studies have been performed over decades for investigation for pharmacological effects of polyphenols, suggesting benefit for health. Growing evidence has shown that a few polyphenols possess anti-inflammatory properties [6–8]. In addition, recent studies have demonstrated that several kinds of polyphenols can inhibit regulatory enzymes or transcription factors which are involved in inflammation [9].

Diet influences different stages of inflammation and demonstrates an important impact on several inflammatory diseases [10, 11]. Inflammation plays a key role on the development of diseases such as diabetes, asthma, cardiovascular diseases, and cancer [12]. Therefore, controlling inflammation may prevent these diseases. Since macrophages are major mediators of inflammation, they have diverse functions in tissue homeostasis and inflammation. Thus, agents effectively modulating their functions are of great clinical value since aberrant activation of macrophages can cause detrimental immune responses. Current research tendency clearly indicates that natural products are one of the most important sources of new drugs [13].

One polyphenol, fisetin (3,3′,4′,7-tetrahydroxyflavone), is contained richly in strawberry, apple, tomato, onion, orange, peach, grape, kiwi, persimmon, tea, and wine [14]. So far, fisetin has reported strong anti-inflammatory [15–17], antioxidant [18], antitumorigenic [19, 20], antiangiogenic [21], antidiabetic [22], neuroprotective [23], and cardioprotective effects [24] both in cell culture and in animal models relevant to human diseases. Although fisetin has many physiological effects, its effect on macrophages is not much reported.

In the present study, we investigated the beneficial effects of fisetin on the inflammatory response of macrophages activated by lipopolysaccharide (LPS).

## 2. Materials and Methods

### 2.1. Isolation of Resident Peritoneal Macrophages

BALB/c mice were obtained from the Institute of Medical Science of the University of Tokyo and maintained in the Animal Resource Center of Okayama University. All the animal experiments were performed to conform to the NIH guidelines (Guide for the Care and Use of Laboratory Animals). The experimental protocol was approved by the Ethics Review Committees for Animal Experimentation of Okayama University Graduate School of Medicine, Dentistry, and Pharmaceutical Sciences (OKU-2018449). All mice were maintained in a barrier facility and fed a normal laboratory diet. Male and female BALB/c mice, 16 ± 4 weeks of age were anesthetized with isoflurane and euthanized via cervical dislocation. Macrophages were harvested via peritoneal lavage using saline (5 mL). The peritoneal lavage solution was centrifuged at 1500 rpm for 5 minutes. The sediment was suspended in a little DMEM (SIGMA, Cat. No. D6046). Red blood cells were lysed using a solution of ammonium chloride. The number of macrophages were calculated using a hemocytometer, then resuspended in DMEM containing heat-inactivated FBS (10% *v*/*v*), penicillin (10 U/mL), and streptomycin (10 mg/mL), and plated into 6-well plates (Corning Incorporated, Cat. No. 3516) [25, 26].

### 2.2. Cell Culture Conditions

After overnight serum starvation, macrophages were incubated with selected concentrations of fisetin (10, 30, and 100 *μ*M; SIGMA, Cat. No. F4043) or vehicle for 3 hours and incubated in the presence or absence of LPS (10 ng/mL; SIGMA, Cat. No. L4391) for an appropriate time. Then, the medium and cells were collected. The medium was used for the nitric oxide (NO) assay and gelatin zymography. Cell lysates were used for quantitative polymerase chain reaction (qPCR) and western blotting.

### 2.3. Real-Time Polymerase Chain Reaction

mRNAs were extracted from macrophages using RNeasy Mini Kits (QIAGEN, Cat. No. 74104). Reverse transcription was performed using an iScript cDNA Synthesis Kit (Bio-Rad, Cat. No. 1708891). qPCR was performed with an ABI Step One Real-Time PCR System (Applied Biosystems, QuantStudio 3) using a Fast SYBR Green Real-time PCR Mixture (Applied Biosystems, Cat. No. 4385612) [27]. Primers for monocyte chemotactic protein 1 (MCP-1) (Takara Bio Inc., Cat. No. MA066003), interleukin 1 beta (IL-1*β*) (Takara Bio Inc., Cat. No. MA025939), inducible nitric oxide synthase (iNOS) (Takara Bio Inc., Cat. No. MA083369), matrix metalloproteinase- (MMP-) 2 (Takara Bio Inc., Cat. No. MA127110), MMP-9 (Takara Bio Inc., Cat. No. MA031311), and 18S ribosomal RNA (18S) (Takara Bio Inc., Cat. No. MA050364) were commercially available. Each sample was normalized to values for 18S mRNA expression (*ΔΔ*CT method) [27].

### 2.4. Nitric Oxide Assay

The individual cell culture supernatants were collected and centrifuged 10 min at 14,000 rpm at 4°C. Then, the supernatant was used for nitric oxide (NO) assay. The NO assay was performed as described in the commercially available kits (BioAssay Systems, Cat. No. D2NO-100). BioAssay Systems' QuantiChrom™ Nitric Oxide Assay Kit is a colorimetric assay for the determination of NO production following reduction of nitrate to nitrite using the improved Griess method.

### 2.5. Western Blotting

Whole cell proteins were extracted from macrophages using a lysis buffer (Cell Signaling, Cat. No. 9803). Each sample was applied into 10% SDS-PAGE and transferred to a polyvinylidene fluoride membrane, immunoblotted with primary antibodies (the phosphorylation of extracellular signal-regulated kinase (p-ERK); Cell Signaling, Cat. No. 9101, the phosphorylation of c-Jun N-terminal kinase (p-JNK); Cell Signaling, Cat. No. 9251, the urokinase plasminogen activator (uPA); Abcam, Cat. No. ab20789, the urokinase plasminogen activator and receptor (uPAR); R&D, Cat. No. AF534, and *β*-actin; Sigma Cat. No. A5441) [28]. Membranes were then incubated with appropriate secondary antibodies, and immune complexes were visualized on chemiluminescence (Merck Millipore, Cat. no. WBLUF0100, Cat. no. WBLUC0100) and quantified using a General Electric Imager (GE Healthcare, LAS 4000 mini) [29].

### 2.6. Gelatin Zymography

Cell culture supernatants were resolved under nonreducing conditions by SDS-PAGE (10% wt/vol) polymerized in the presence of gelatin (0.1% wt/wt) to evaluate the activities of MMP-9. Gels were washed with Triton X-100 (2.5% vol/vol) for 60 min and distilled water for 15 min two times. Gels were then incubated overnight at 37°C in 50 mM Tris buffer containing calcium chloride (5 mM) pH 8.0. After incubation, gels were stained with Coomassie Brilliant Blue R-250 (Bio-Rad, Cat. No. 161-0436) for 30 min at room temperature, then followed by destaining with Coomassie Brilliant Blue R-250 Destaining Solution (Bio-Rad, Cat. No. 161-0438) until clear bands appeared. Gel images were quantified using a General Electric Imager (GE Healthcare, LAS 4000 mini); the unstained, translucent digested regions represented areas of MMP activity [30].

### 2.7. Statistics

All statistical analyses were performed using Sigma Plot v14.0 (Systat Software Inc., California, USA). Data are presented as the mean ± standard error of the mean. Statistical significance between multiple groups was assessed by one-way or two-way analysis of variance followed by Holm-Sidak post hoc test or Student-Newman-Keuls post hoc test. *p* value < 0.05 was considered statistically significant.

## 3. Results

### 3.1. Fisetin Attenuated Inflammatory Mediators in Macrophages Stimulated with LPS

**Figure 1:**
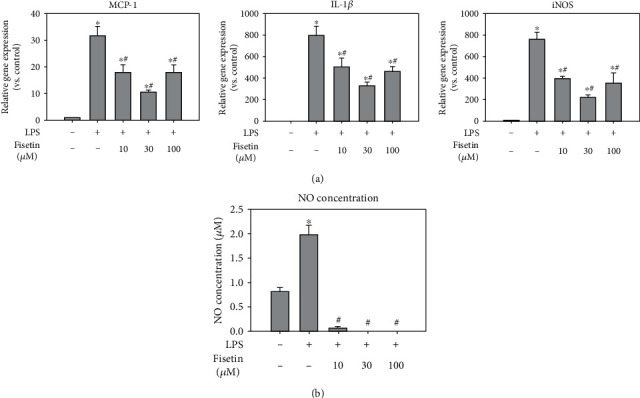
Effects of fisetin on the expression of inflammatory mediators in macrophages stimulated with LPS. (a) mRNA expression of MCP-1, IL-1*β*, and iNOS in LPS- (10 ng/mL) stimulated macrophages in the presence or absence of fisetin (10-100 *μ*M) by qPCR (*n* = 6 in each group). Each bar presents the mean + SE of six experiments. ^∗^*p* < 0.05 vs. control group; ^#^*p* < 0.05 vs. LPS-stimulated group. (b) NO concentration in each cell culture supernatants by colorimetric assay (*n* = 3 in each group). Each bar presents the mean + SE of three experiments. ^∗^*p* < 0.05 vs. control; ^#^*p* < 0.05 vs. LPS.

Since activated macrophages produce proinflammatory cytokines and inflammatory mediators such as NO [8], we examined the effects of fisetin on inflammatory mediators in macrophages stimulated with LPS. First, to study whether fisetin exerts the anti-inflammatory effect at the transcriptional level, we determined mRNA expression levels of inflammatory genes such as MCP-1, IL-1*β*, and iNOS (Figure 1(a)). As expected, the expressions of these inflammatory genes were suppressed by fisetin in a dose-dependent manner. Next, to confirm whether fisetin suppresses inflammatory response in macrophages, we evaluated its capability to suppress NO production in LPS-treated macrophages (Figure 1(b)). As expected, fisetin decreased production of NO in a dose-dependent manner.

### 3.2. Fisetin Reduced Mitogen-Activated Protein Kinase in Macrophages Stimulated with LPS

**Figure 2:**
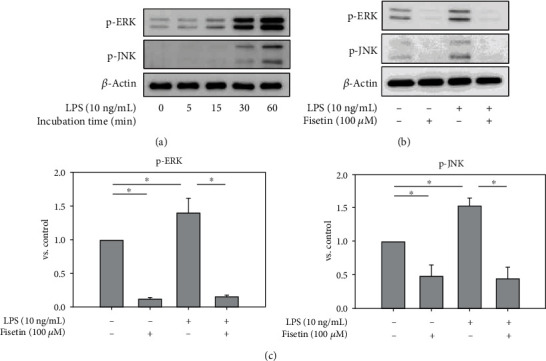
Effects of fisetin on the protein expression of MAPKs in macrophages stimulated with LPS. (a) Protein expression of MAPK in LPS- (10 ng/mL) stimulated macrophages for 0-60-minute incubation (*n* = 3 in each group). (b) Protein expression of MAPK in LPS- (10 ng/mL) stimulated macrophages in the presence or absence of fisetin (100 *μ*M) by western blotting (*n* = 3 in each group). (c) The relative expression of each protein was quantified by densitometry and normalized to the band under the control condition. Each bar presents the mean + SE of three experiments. ^∗^*p* < 0.05.

Since fisetin was reported to inhibit mitogen-activated protein kinase (MAPK) pathways in various cell types [31, 32], we examined the effects of fisetin on MAPKs in macrophages. First, activation of MAPKs induced by LPS in macrophages was observed (Figure 2(a)). The increase in p-ERK by LPS peaked at 15 minutes after stimulation, while the increment of p-JNK by LPS peaked at 30 minutes. Thus, in the subsequent experiments, macrophages were stimulated with LPS for 30 minutes. Cotreatment with fisetin significantly decreased LPS-induced enhancement of p-ERK and p-JNK (Figures 2(b) and 2(c)).

### 3.3. Fisetin Reduced uPA and uPAR in Macrophages Stimulated with LPS

**Figure 3:**
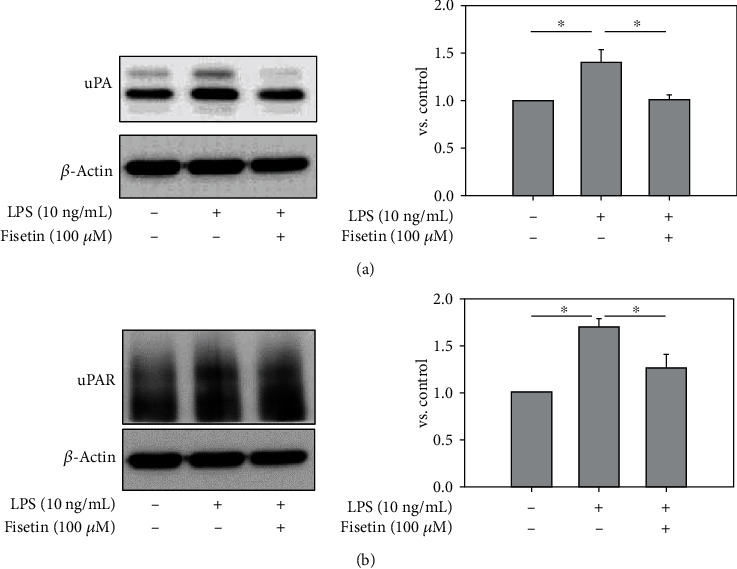
Effects of fisetin on the protein expression of uPA and uPAR in macrophages stimulated with LPS. (a) Protein expression of uPA in LPS- (10 ng/mL) stimulated macrophages in the presence or absence of fisetin (100 *μ*M) by western blotting. The relative expression of each protein was quantified by densitometry and normalized to the band under the control condition (*n* = 3 in each group). Each bar presents the mean + SE of three experiments. ^∗^*p* < 0.05. (b) Protein expression of uPAR in LPS- (10 ng/mL) stimulated macrophages in the presence or absence of fisetin (100 *μ*M) by western blotting. The relative expression of each protein was quantified by densitometry and normalized to the band under the control condition (*n* = 3 in each group). Each bar presents the mean + SE of three experiments. ^∗^*p* < 0.05.

Macrophage activation is closely correlated to the invasiveness of cells. In addition, uPA and uPAR in macrophages contribute to the infiltration of cells. Thus, the effect of fisetin on the expression of uPA and uPAR was investigated. LPS induced the protein abundance of uPA (Figure 3(a)) and uPAR (Figure 3(b)). Cotreatment of fisetin reduced the increment of uPA (Figure 3(a)) and uPAR by LPS (Figure 3(b)).

### 3.4. Fisetin Reduced MMP-2 and MMP-9 in Macrophages Stimulated with LPS

**Figure 4:**
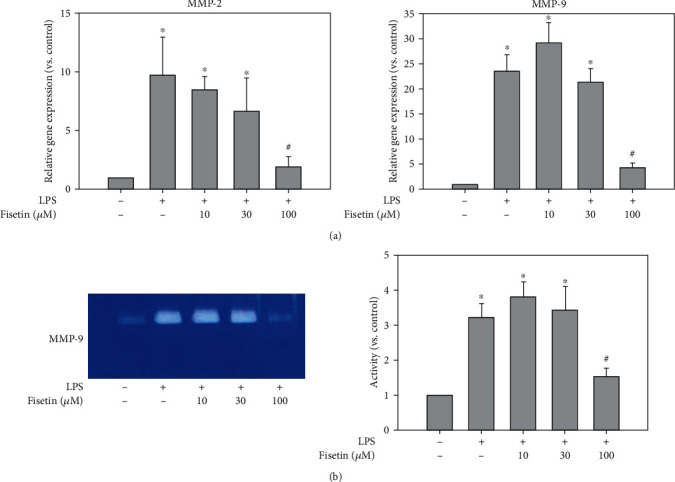
Effects of fisetin on MMP-2 and MMP-9 in macrophages stimulated with LPS. (a) mRNA expression of MMP-2 and MMP-9 in LPS- (10 ng/mL) stimulated macrophages in the presence or absence of fisetin (10-100 *μ*M) by qPCR (*n* = 6 in each group). Each bar presents the mean + SE of six experiments. ^∗^*p* < 0.05 vs. control group; ^#^*p* < 0.05 vs. LPS-stimulated group. (b) The activation of MMP-9 in LPS- (10 ng/mL) stimulated macrophages in the presence or absence of fisetin (10-100 *μ*M) by gelatin zymography. The relative expression of MMP-9 activity was quantified by densitometry and normalized to the band under the control condition (*n* = 3 in each group). Each bar presents the mean + SE of three experiments. ^∗^*p* < 0.05.

Furthermore, to elucidate effect of fisetin on MMPs, we examined MMP-2 and MMP-9 expressions, the downstream mediators of uPA/uPAR. Regarding the mRNA level, LPS significantly increased MMP-2 and MMP-9 mRNA expression, compared with the vehicle treatment (Figure 4(a)). Fisetin reduced the enhancement of these gene expressions of MMP-2 or MMP9 induced by LPS in a dose-dependent manner (Figure 4(a)). Moreover, the protein activities of MMP-9 were evaluated by zymography (Figure 4(b)). The protein activities of MMP-9 (92 kDa) were increased by LPS stimulation, which was reduced by fisetin (Figure 4(b)).

## 4. Discussion

We found an important effect of fisetin against inflammation in macrophages. The present study demonstrated that fisetin attenuated the induction of inflammatory components such as MCP-1, IL-1*β*, iNOS, and NO, induced by LPS in macrophages. In addition, fisetin suppressed the activation of MAPK and attenuated the LPS-induced increment of uPA and uPAR expression. Moreover, fisetin attenuated the increase of mRNA abundance of MMP-2 and MMP-9 and the activity of MMP-9.

In sepsis caused by gram-negative infection, LPS is commonly recognized as the causative agent of sepsis. In many studies, LPS has been used as a model of sepsis as well as an inflammation. Moreover, chronic inflammation induces early cancer by activation of inflammatory cells and improper production of a preinflammatory mediator like iNOS and transcription factors like the nuclear transcription factor kappa B (NF-*κ*B) [33]. Besides, it has also been reported that oxidative stress is reduced by attenuating the expression of iNOS [34]. Our study demonstrated that fisetin may be effective in the prevention and treatment of sepsis and septic shock, due to the suppressive effect on mRNA abundance of proinflammatory genes and the production of NO. In this study, fisetin suppressed NO production by reducing the expression of mRNA of the proinflammatory gene (iNOS); therefore, it was likely that fisetin attenuated the oxidative stress. Consequently, these results supported the anti-inflammatory, antitumorigenic, and antioxidant effects of fisetin.

Several studies have reported that LPS induced the phosphorylation of MAPKs, including ERK and JNK [35]. In addition, it has been reported that fisetin inhibits migration and invasion of human cervical cells by downregulating uPA expression through suppressing MAPK [36]. In macrophages, few reports are available regarding the effect of fisetin on the expression and activity of uPA and uPAR. Our study demonstrated for the first time that fisetin suppressed the expression of uPA and uPAR induced by LPS through suppressing MAPK signaling in macrophages, as previously reported in cancer cells.

MMPs are a family of proteases whose expression is related to certain processes, such as development, physiology, and pathology. In common, MMP-9 has been recognized as a marker of various inflammatory diseases, such as atherosclerosis, arthritis, and systemic lupus erythematosus [37]. In addition, MMP-9 was closely associated with cell migration [38, 39]. Several studies have particularly reported that MMP-2 and MMP-9 are involved in atherogenesis and play an important role in the stabilization of atherosclerotic plaques [40–42]. On this point, our results suggest that inhibitors of MMP-9 can offer promise as a therapeutic approach for the stabilization of vulnerable plaques. Moreover, recent investigations have demonstrated that wound healing in the skin is impaired by excessive activation of MMP-9 [43]. Therefore, it was suggested that fisetin also has potential as a therapeutic approach for healing in the skin. Future studies will be required to elucidate its detail on molecular mechanism.

A recent trend in the pharmaceutical industry is discovering strategies to develop the natural plant itself as the new drug. Indeed, two recent animal studies have demonstrated that LPS-induced acute pulmonary inflammation and collagen-induced arthritis were improved by fisetin treatment [32, 44]. Another *ex vivo* study also demonstrated that fisetin was able to attenuate LPS-induced cytokine release from leukocytes of patients with chronic obstructive pulmonary disease or type 2 diabetes [45]. Many immunosuppressive drugs have been developed to attenuate inflammatory and autoimmune diseases [46–50]; nevertheless, most compounds exhibit significant side effects. Since polyphenols are commonly present in dietary fruits and vegetables, they presumably may be safer immunosuppressive agents [51, 52]. Fruits and vegetables contain a wide array of phytochemicals including sesquiterpene lactones, terpenoids, polysaccharides, and phenolic compounds. Our results, demonstrating the anti-inflammatory, antitumorigenic, and antioxidant effects of fisetin, strongly indicate that it can be developed as a new drug. Although fisetin is a promising candidate for immunotherapy, the potential of natural products containing fisetin as a therapeutic agent for a certain disease needs to be further verified in the clinical setting.

## 5. Conclusions

The present study demonstrated that fisetin attenuated the increments of inflammatory genes (MCP-1, IL-1b, and iNOS), the production of NO, and the phosphorylation of MAPK, uPA, uPAR, and MMPs, induced by LPS in macrophages. These results suggested that fisetin may be a therapeutic agent for several macrophage-related diseases.

## Data Availability

All material and data are present in the manuscript.
